# Supplementary material for: Repurposing β-Lactams for the Treatment of Mycobacterium kansasii Infections: An In Vitro Study
Source: Antibiotics (Basel). 2023 Feb 5;12(2):335. doi: 10.3390/antibiotics12020335 (PMC9952313; doi:10.3390/antibiotics12020335)

Repurposing β-Lactams for the Treatment of *Mycobacterium kansasii* Infections: An In Vitro Study

Lara Muñoz-Muñoz, José A. Aínsa and Santiago Ramón-García

Figure S1. Time kill-assays of rifampicin and ethambutol (backbone therapy drugs) in combination with amoxicillin/clavulanate, cefadroxil, isoniazid and clarithromycin against M. kansasii ATCC 12478. Time-kill assays were performed in Middlebrook 7H9 broth plus ADC. MIC values used were: AMX/CLV: 8 mg/L; RIF: 0.125 mg/L; EMB: 4 mg/L; CLA: 0.25 mg/L and INH: 8 mg/L. Clavulanate was added at a fixed dose of 4mg/L. AMX/CLV: amoxicillin/clavulanate; CFX: cefadroxil; RIF: rifampicin; EMB: ethambutol; INH: isoniazid; CLA: clarithromycin.

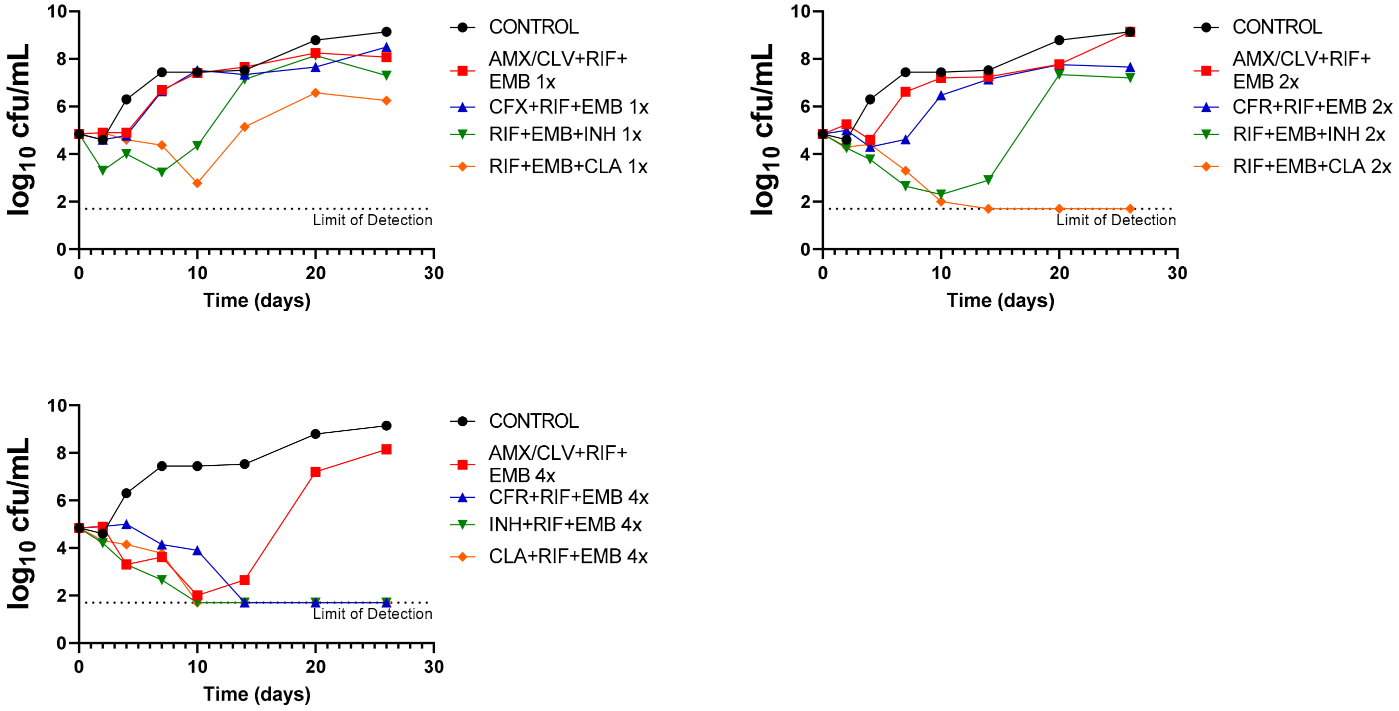

Supplement: Supplementary file 1 [file antibiotics-12-00335-s001.zip › antibiotics-2162038_Supplementary data (2).pdf]
